# The diagnostic efficacy of venous-phase spectral CT combined with Node-RADS for differentiating enlarged lymph nodes in common solid tumors: a preliminary exploratory study

**DOI:** 10.3389/fonc.2026.1840436

**Published:** 2026-07-08

**Authors:** Xia Su, Hanhong Xie, Jiatong Liang, Xingxing Xie, Lan Lan, Peng Peng

**Affiliations:** 1 Department of Radiology, The First Affiliated Hospital of Guangxi Medical University, Nanning, China; 2 NHC Key Laboratory of Thalassemia Medicine, Guangxi Medical University, Nanning, China

**Keywords:** 100 keV, combined approach, lymph node metastasis, Node-RADS, quantitative parameters, spectral CT, venous phase

## Abstract

**Objective:**

To investigate the value of combining quantitative parameters from spectral CT with the Node Reporting and Data System 1.0 (Node-RADS) in distinguishing between benign and malignant lymph nodes enlarged due to solid tumors, and to explore a combined multiparametric approach.

**Methods:**

We retrospectively analyzed data from patients with primary solid tumors accompanied by lymphadenopathy who underwent venous-phase spectral CT scans with energy-resolved imaging. A total of 243 lymph nodes were included in this study. Based on pathological findings, clinical observations, and CT follow-up, the patients were divided into malignant (n = 124) and benign (n = 119) groups. The groups were compared for general characteristics. For each lymph node, we measured the venous-phase spectral CT parameters, including iodine concentration (IC), normalized iodine concentration (NIC), effective atomic number (Z_eff_), and CT values of virtual monoenergetic images (VMI) at different energy levels (40 keV,70 keV, 100 keV). We also calculated the spectral curve slope (λ) for different energy ranges. A node-RADS score was assigned to each lymph node. Variables were screened using univariate and multivariate logistic regression analyses and applied to develop a combined exploratory approach. The model’s diagnostic performance was evaluated using receiver operating characteristic (ROC) curves. The differences in the area under the curve (AUC) values between the two models were assessed using the DeLong test. Calibration analysis and bootstrap internal validation were also performed.

**Results:**

The malignant and benign groups showed significant differences (*P* < 0.05) in Node-RADS scores, Z_eff_, CT values for VMI (40 keV, 70 keV, 100 keV), λ, and IC. Based on the multivariate logistic regression analysis, the final multivariable regression model included the CT value at 100 keV on VMI (*OR* = 0.970, 95% confidence interval [CI]: 0.949–0.991, *P* = 0.005) and Node-RADS score (*OR* = 3.856, 95% CI: 2.666–5.576, *P* = 0.001). At a cutoff value of 0.495, the AUC, sensitivity, specificity, and accuracy of the combined model for identifying metastatic lymph nodes were 0.819 (95% CI: 0.766–0.872), 72.6% (95% CI: 0.638–0.802), 78.2% (95% CI: 0.696–0.853), and 75.3% (95% CI: 0.694–0.806), respectively. The DeLong test revealed significant differences in the AUCs between the combined model and the single Node-RADS model for differentiating the benign or malignant nature of lymph nodes (*Z* = 2.138, *P* = 0.033). Bootstrap validation confirmed model stability, and the combined model demonstrated good calibration (Brier score = 0.173).

**Conclusion:**

An exploratory approach combining quantitative venous-phase spectral CT (VMI _100 keV_) parameters with Node-RADS scoring may provide a modest statistical improvement when identifying whether enlarged lymph nodes are benign or malignant. These preliminary exploratory observations suggest an objective imaging approach that warrants further prospective and multicenter validation, alongside formal clinical utility analyses, before considering routine clinical implementation.

## 1 Introduction

Accurate differentiation between benign and malignant lymph nodes is critical for accurate tumor staging, treatment planning, and prognosis assessment (1, 2). Incorrect staging can lead to unnecessary lymph node dissection and biopsy or missed diagnoses of metastasis, resulting in a poor prognosis. The Node Reporting and Data System (Node-RADS) provides a standardized framework that enhances inter-observer agreement and streamlines clinical communication. Growing evidence supports its potential to standardize radiological reporting across a variety of solid malignancies (3, 4). However, it lacks sufficient diagnostic specificity for atypical lymph nodes, such as those with micrometastases without necrosis or inflammatory hyperplasia, and its use alone has significant limitations. Spectral CT is noninvasive and provides a variety of quantitative parameters that reflect tissue blood supply, composition, and microstructure (5, 6), offering additional evidence for assessing lymph node metastasis in solid tumors. Numerous studies have demonstrated the high clinical utility of spectral CT in distinguishing between benign and malignant tumors, determining histological subtypes, staging, and evaluating treatment efficacy (7, 8). Currently, clinical practice relies primarily on single-modality imaging assessments, and research exploring whether quantitative parameters derived from spectral CT can provide complementary quantitative information for the standardized NodeRADS (9) is scarce. In this study, we integrated Node-RADS 1.0 with multiple quantitative spectral CT parameters to construct a combined exploratory approach. Using this multivariable regression model, we retrospectively evaluated the diagnostic performance of spectral CT parameters for distinguishing between benign and malignant lymph nodes enlarged due to solid tumors during the venous phase. By exploring this combined approach, we aim to provide preliminary proof-of-concept evidence for a noninvasive imaging tool that requires further formal clinical utility analyses before it can be applied to preoperative risk stratification and treatment decision-making.

## 2 Materials and methods

### 2.1 Research data

**Figure 1:**
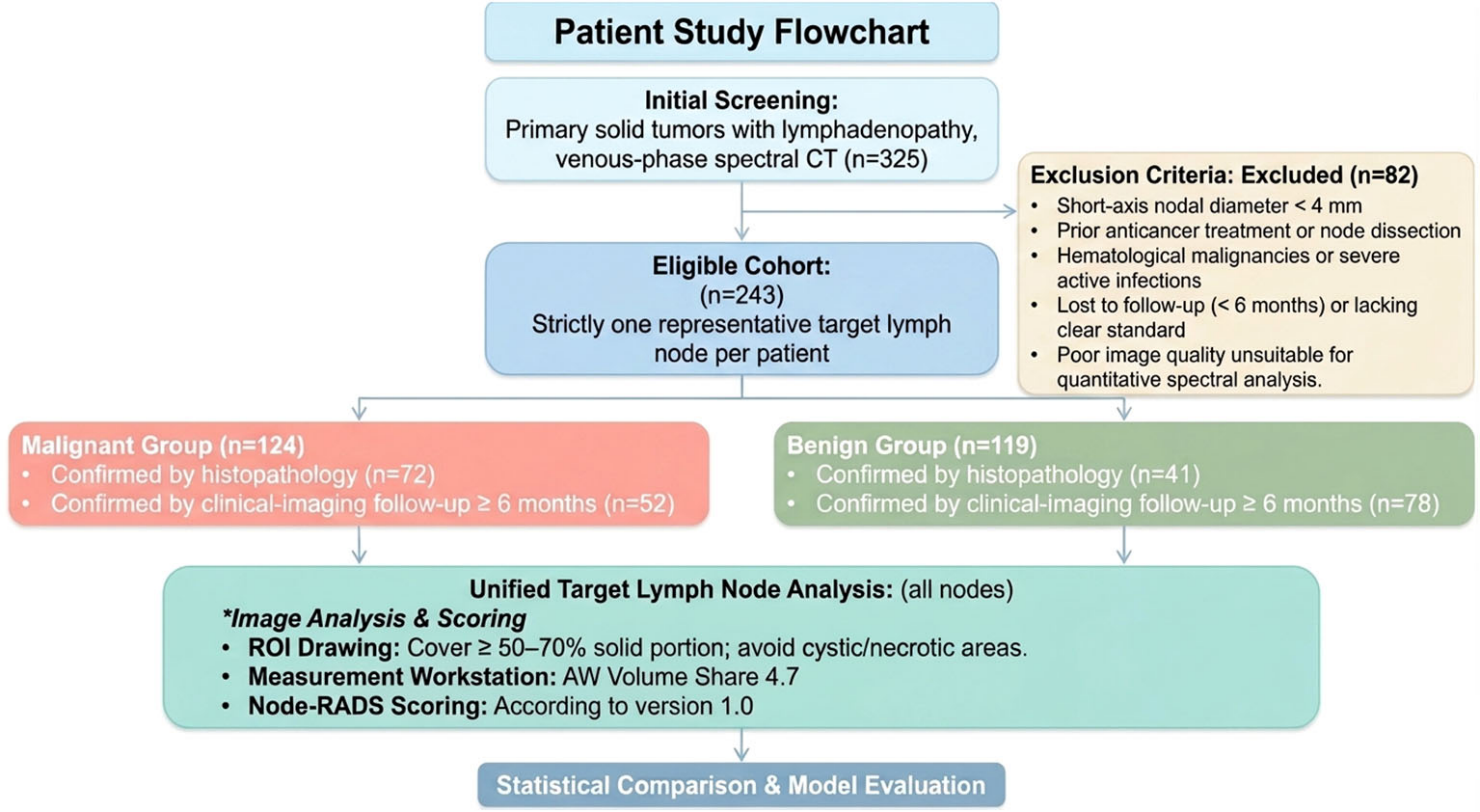
Patient flow diagram. Shows patient screening, exclusion criteria, and the final cohort of 243 target lymph nodes stratified by reference standard and diagnosis.

We included 243 patients with lymphadenopathy attributable to solid tumors who underwent venous-phase spectral CT at our medical center between January 2023 and December 2025. The inclusion criteria were (1): pathologically confirmed primary malignant solid tumors, with focus on lung, liver, colorectal, and breast malignancies to maintain representative venous-phase enhancement; and (2) measurable target lymph nodes on CT images (initial screening threshold ≥ 5 mm; a few borderline nodes measuring 4 mm during blinded re-evaluation were retained to avoid post-hoc exclusion bias). Benign and malignant lymph nodes were distinguished based on one of the following two criteria: (a) confirmed lymph node pathology results obtained through lymph node fine-needle aspiration biopsy or surgical dissection, and (b) well-defined primary tumor pathology along with 6–12 months of continuous imaging and clinical data that definitively determined its benign or malignant nature, in cases where the lymph node pathology was not available. Specifically, these patients underwent at least three consecutive contrast-enhanced CT examinations spaced more than 3 months. Patients who received anticancer treatment before the spectral CT scan and/or lymph node dissection were excluded. We also excluded patients with lymphoma, leukemia, or severe active infections, and those with poor-quality images that did not meet the requirements for measuring quantitative spectral parameters. The detailed patient selection process is illustrated in **Figure 1**. This study was conducted in accordance with the principles of the Declaration of Helsinki and approved by the Ethics Committee of the First Affiliated Hospital of Guangxi Medical University (2026-E0236).

### 2.2 Matching target lymph nodes between images and reference standards

The target lymph nodes were selected exclusively from the regional lymphatic drainage basins of the primary tumor, and were evaluated based on their anatomical regions and integrated pathological and clinical feedback. The anatomical regions of the lymph nodes throughout the body were defined according to relevant international standards. Cervical lymph nodes were classified according to radiological staging criteria (10), thoracic lymph nodes according to the International Association for the Study of Lung Cancer (IASLC) atlas (11), and abdominal and pelvic lymph nodes were localized based on major blood vessels and regional anatomical landmarks (12). For patients with multiple enlarged lymph nodes, the node with direct biopsy confirmation was prioritized. When multiple nodes were present within a regional station confirmed by pathology or longitudinal follow-up, the largest node (based on short-axis diameter) was selected to minimize selection bias. The target lymph nodes were matched using one of the following methods: (a) direct matching based on CT landmarks and biopsy procedural records (biopsy-confirmed), (b) station-based index node matching by designating a representative “index node” for each station, to bridge the gap between station-based pathology and specific CT lesions. For confirmed metastatic stations, the largest node was selected as the malignant target, whereas for negative stations, the largest node on the CT scan was designated as a benign target. (c) Clinical-imaging follow-up: For cases without pathology, we required ≥ 6 months of follow-up. Lesions that showed significant volume progression or development of new metastases were classified as malignant, whereas those that shrank or remained stable following anti-inflammatory therapy or primary tumor treatment were classified as benign.

### 2.3 CT acquisition

To optimize the thermal load of the X-ray tube while maintaining image interpretability across multiple phases, we employed conventional modes for the arterial and delayed phases and energy-spectrum scanning mode for the venous phase. Given that contrast agent concentrations are most stable in the venous phase within the blood vessels and target lymph nodes, the quantitative spectral analysis was conducted based on spectral data from this phase. All enrolled patients underwent 256-slice spectral CT (Revolution CT; GE Healthcare, Milwaukee, WI, USA) covering the neck, chest, abdomen, and pelvis in a head-to-toe orientation. Acquisition during the venous phase was performed using the Gemstone Spectral Imaging (GSI) mode. Rapid transient kV switching technology was used at 80/140 kVp, tube rotation speed was 0.5/s, pitch was 0.992:1, scan field of view (SFOV) was 50 cm, display field of view (DFOV) was 40 × 40 cm, detector collimation was 80 mm, and both reconstruction slice thickness and slice spacing were 1.25 mm. A contrast-enhanced scan was performed via the patient’s median cubital vein. The contrast agent iopamidol (350 mg/mL, 1 mL/kg) was administered using a double-chamber syringe at 3.5 mL/s, followed by injection of normal saline (20 mL) at the same rate. Scanning was triggered using in-plane dynamic monitoring technology with the region of interest positioned within the thoracic aorta below the level of the bronchial bifurcation. The CT value trigger threshold was set at 180 HU, and a 90-second delay was applied before performing the venous-phase scan once the threshold was reached. Spectral images were post-processed on an AWVolume Share 4.7 workstation, and GSI Viewer was utilized to quantify the relevant spectral parameters for each lesion.

### 2.4 Data measurement and analysis

The ROI was drawn as large as possible to cover at least 50–70% of the solid portion of the lymph node, strictly avoiding cystic, necrotic, or calcified areas. Two non-consecutive representative slices were selected; the ROI was delineated and measured, and the average was calculated. We measured the CT values of the virtual monoenergetic images at 40, 70, and 100 keV, energy spectrum slope (λ _40–70 keV_, λ _70–100 keV_, λ _40–100 keV_), iodine concentration (IC), normalized iodine concentration (NIC), normalized water concentration (NWC), and effective atomic number (Z_eff_). For NIC and NWC calculations, a circular ROI was placed in the aortic lumen at the same slice level. For pelvic or inguinal nodes where the aorta could not be visualised, the ROI was placed on the nearest adjacent slice containing the distal abdominal aorta or common iliac artery, while avoiding calcifications or thrombi. Using the predefined composite reference standard, we analyzed the differences in quantitative parameters between benign and malignant lymph nodes using the following equation:


$$\lambda_{E_{L}}{-}_{E_{H}}=\frac{HU_{E_{L}}-HU_{E_{H}}}{E_{H}-E_{L}}$$

where E_L_ and E_H_ represent the low and high energy levels, respectively. In this study, quantitative analysis was performed for three energy ranges: 40–70 keV, 70–100 keV, and 40–100 keV. Two physicians with more than five years of experience in CT imaging diagnosis evaluated the morphological characteristics of the enrolled lymph nodes using a blinded evaluation method, and scoring was performed according to Node-RADS 1.0. In cases of disagreement, the two parties reached a consensus after discussion.

### 2.5 Statistical methods

Statistical analyses and graphing were performed using SPSS (version 26.0) and GraphPad Prism 10.1.2 (GraphPad Software Inc., San Diego, CA, USA). Quantitative data were first tested for normality using the Kolmogorov-Smirnov test. Data that followed a normal distribution were expressed as mean ± standard deviation, and comparisons between groups were performed using an independent samples t-test. Data that did not follow a normal distribution were described as median (Q1, Q3) and compared using the Mann-Whitney U test. Before conducting multivariable modeling, multicollinearity among significant variables was assessed using the variance inflation factor (VIF). Given the low-dimensional nature of our predefined variables, a VIF-based iterative feature-selection strategy was preferred over penalized regression (e.g., LASSO) to ensure clinical interpretability and explicitly retain the core Node-RADS score. To prevent model instability caused by highly correlated spectral parameters (VIF > 10), an iterative feature-selection strategy was applied, and only independent predictors were retained in the final multivariable binary logistic regression model. The diagnostic performance of the models was evaluated using receiver operating characteristic (ROC) curves and the area under the curve (AUC), by calculating the model’s sensitivity, specificity, agreement rate, and calibration. The optimal cutoff values for the logistic regression models were determined by maximizing the Youden index. The DeLong test was used to compare the AUCs of the two models. Statistical significance was set at p< 0.05. Internal validation was performed using bootstrap resampling with1,000 resamples. Model calibration was assessed using the Hosmer–Lemeshow test, Brier score, and calibration curves.

## 3 Results

### 3.1 Patient demographics

A total of 243 patients (166 men and 77 women; median [range] age: 60 [8-89] years; mean age:59.1 years) were included in this study. To avoid statistical clustering effects, exactly one representative target lymph node was selected per patient, resulting in 243 target lymph nodes for analysis. Based on the reference standard, the cohort was divided into malignant (n = 124) and benign (n = 119) groups. There were no statistically significant differences between the two groups in age, gender, and the distribution of primary tumor types (*P* > 0.05). The baseline clinical, demographic, and primary tumor distribution data for all patients are summarized in **Tables 1**, **2**. The data measurements were evaluated for reproducibility. Patient names and other identifying information were excluded from the analysis. A subset of 40 randomly selected lymph nodes was independently re-evaluated by a second experienced radiologist. To assess intraobserver reproducibility, the measurements were repeated by the same radiologist after a one-month interval. The intraclass correlation coefficients (ICCs) for all continuous spectral CT quantitative parameters were greater than 0.85. Furthermore, the interobserver agreement between the two evaluating physicians for ordinal Node-RADS scores was excellent (kappa > 0.80).

**Table 1:** Study participant characteristics of 243 patients.

Characteristics	Mean±SD	Median (Q1,Q3)	Range
Sex			
Male	166		
Female	77		
Group			
Benign	119		
Malignant	124		
Age	59.09 ± 12.97	60 (51,69)	8–89
Short-axis diameter	13.36 ± 7.23	11.5 (9,15.63)	4–65.38
Node-RADS	3.34 ± 1.03	3 (3,4)	1–5
λ40-70kev	3.62 ± 1.29	3.546 (2.759,4.503)	0.522–8.445
λ40-100kev	2.28±0.81	2.250 (1.734,2.838)	0.329–5.317
λ70-100kev	0.94±0.35	0.929 (0.717,1.176)	-0.013–2.445
VMI 40keV	185.32±54.29	181. 500 (144.590,224.220)	48.080–353.105
VMI 70 keV	76.79 ± 18.76	77.545 (64.909,90.520)	27.985–117.95
VMI 100 keV	48.48 ± 12.44	49.755 (40.250,57.135)	10.085-82.340
IC	19.25 ± 6.84	18.995 (14.590,23.955)	2.790–44.850
NIC	0.42 ± 0.16	0.425 (0.313,0.517)	0.055–1.080
NWC	0.994 ± 0.01	0.995 (0.998,1.000)	0.945–1.025
Zeff	8.73 ± 0.36	8.730 (8.485,8.995)	7.755–9.975

IC, iodine concentration; NIC, normalized iodine concentration; NWC, normalized water concentration; SD, standard deviation; VMI, virtual monoenergetic image; Zeff, effective atomic number.

The initial screening threshold was short-axis diameter **≥** 5 mm; a few borderline nodes measuring 4 mm during blinded re-evaluation were retained to avoid post-hoc exclusion bias.

**Table 2:** Distribution of the primary tumor.

Primary tumor type	Benign (n = 119)	Malignant (n = 124)	χ2	P-value
			9.459	0.051
Lung cancer	41 (34.5%)	24 (19.4%)		
Liver cancer	36 (30.3%)	43 (34.7%)		
Colorectal cancer	18 (15.1%)	32 (25.8%)		
Breast cancer	9 (7.6%)	12 (9.7%)		
Others	15 (12.6%)	13 (10.5%)		

### 3.2 Morphological characteristics of lymph nodes

The shortest diameter of the largest lymph node was 10.68 ± 4.38 mm in the benign group and 15.92 ± 8.42 mm in the malignant group. The Node-RADS scores for both groups are presented in **Table 3**.

**Table 3:** Comparison of Node-RADS scores between the two groups.

Node-RADS	Benign	Malignant
1	7 (5.9%)	0 (0%)
2	39 (32.8%)	5 (4.0%)
3	51 (42.9%)	36 (29%)
4	16 (13.4%)	53 (42.7%)
5	6 (5.0%)	30 (24.2%)

### 3.3 Comparison of spectral CT parameters between groups

**Figure 2:**
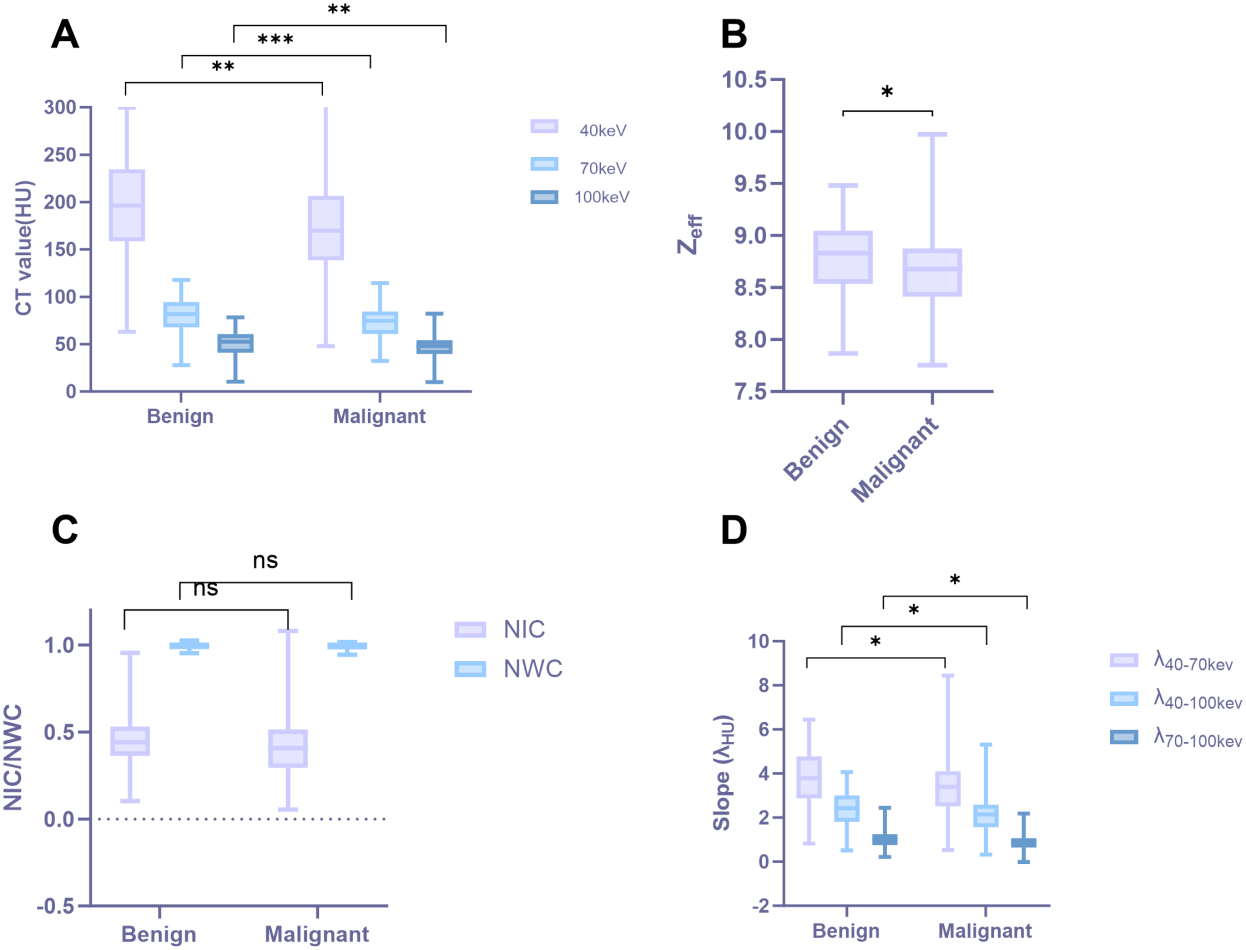
Comparison of spectral CT parameters and clinical features between benign and malignant lymph nodes. **(A–D)** Boxplots illustrating the distributions of **(A)** VMI (40, 70, 100 keV), **(B)** Zeff, **(C)** NIC and NWC, **(D)** spectral curve slopes (λ).

**Figure 3:**
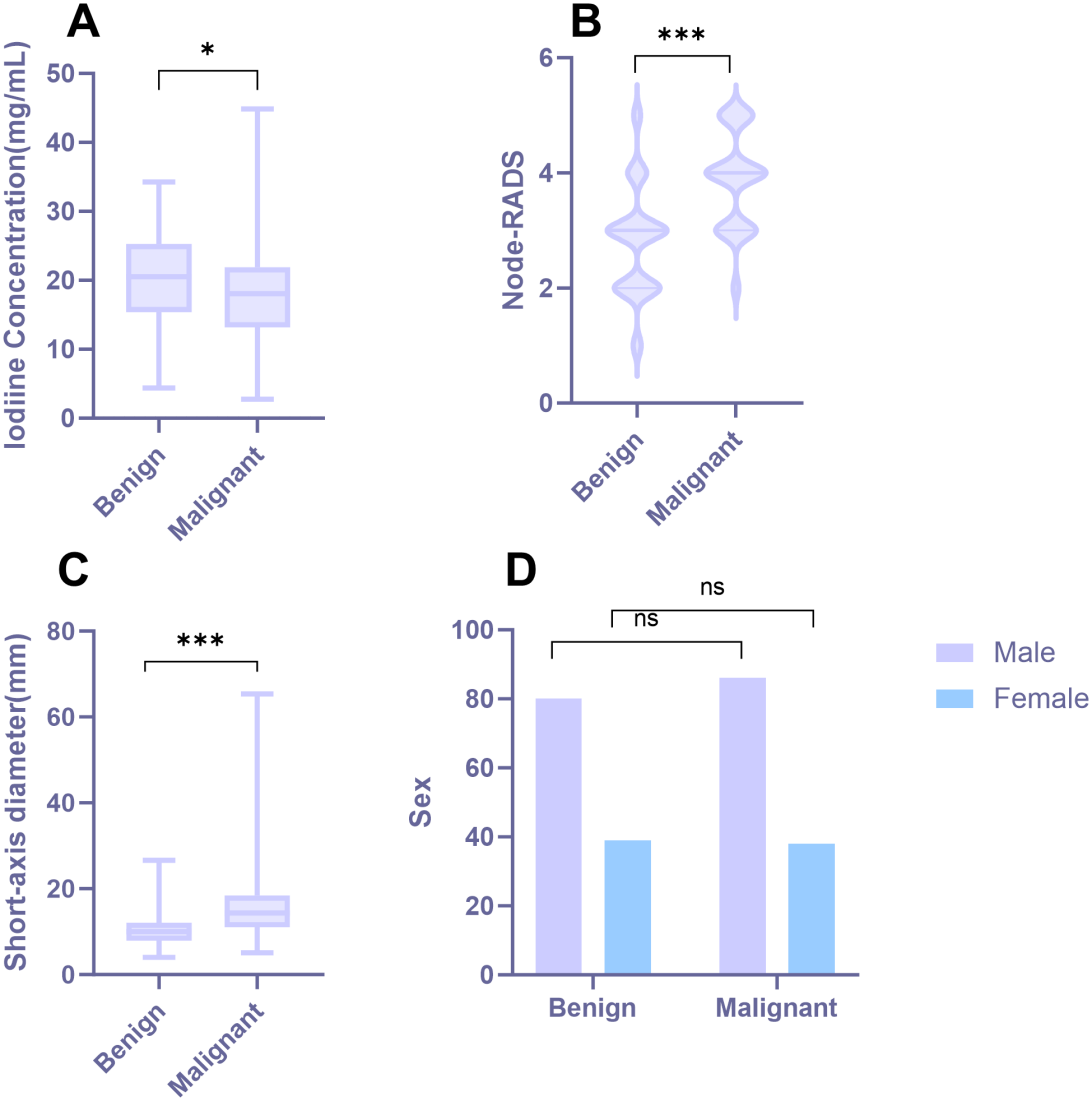
Box plot of iodine concentration **(A)**, violin plot of Node-RADS scores **(B)**, box plot of lymph node short-axis diameter **(C)**, and bar chart of gender distribution **(D)** between benign and malignant groups.

Details of the univariate analysis comparing the spectral CT parameters and Node-RADS scores between the two groups are provided in **Table 4** and illustrated in **Figures 2**, **3**. To determine the independent statistical contributions of these features, significant variables were evaluated using a multivariate logistic regression model (**Table 5**). The differences in Z_eff_, IC, and spectral curve slopes (λ_40–70 keV_, λ_70–100 keV_, λ_40–100 keV_), and CT values corresponding to the 40/70/100 keV virtual monoenergetic images were all statistically significant (*P* < 0.05), whereas the differences in NIC and NWC were not statistically significant (*P* > 0.05).

**Table 4:** Univariate analysis of imaging parameters between benign and malignant lymph nodes.

Characteristics	Z	P-value
Age	-0.410	0.967
Sex	-0.356	0.722
Short-axis diameter	-6.902	<0.001
Node-RADS	-8.315	<0.001
λ40-70kev	-2.410	0.016
λ40-100kev	-2.547	0.011
λ70-100kev	-2.643	0.008
VMI 40 keV	-2.958	0.003
VMI 70 keV	-3.421	0.001
VMI 100 keV	-2.817	0.005
IC	-2.526	0.012
NIC	-1.703	0.089
NWC	-0.825	0.409
Zeff	-2.655	0.008

*Z* represents the statistic from the Mann-Whitney U test.

**Table 5:** Final multivariable binary logistic regression model for differentiating malignant from benign lymph nodes.

Characteristics	β	Standard error	Wald χ2	Odds ratio(95% confidence interval)	P-value
Node-RADS	1.358	0.191	50.544	3.856 (2.666–5.576)	<0.001
VMI 100 keV	-0.034	0.013	6.813	0.970 (0.949–0.991)	0.005

### 3.4 Multivariable logistic regression analysis

After assessing multicollinearity among significant variables, an iterative feature-selection strategy was applied. The final multivariable logistic regression model retained the Node-RADS score and VMI 100 keV as independent predictors. The analysis showed that the corresponding CT values on the 100 keV images (*OR* = 0.970, 95% CI: 0.949–0.991, *P* = 0.005) and Node-RADS scores (*OR* = 3.856, 95% CI: 2.666–5.576, *P* = 0.001) were independent predictors of lymph node benignity or malignancy. Based on these independent predictors, we formulated the following equation for the combined regression model: Logit(P) = -2.858 + 1.358 × Node-RADS - 0.034 × VMI _100 keV._ The predicted probability (PRE) was used as the test variable for subsequent ROC curve analysis.

**Figure 4:**
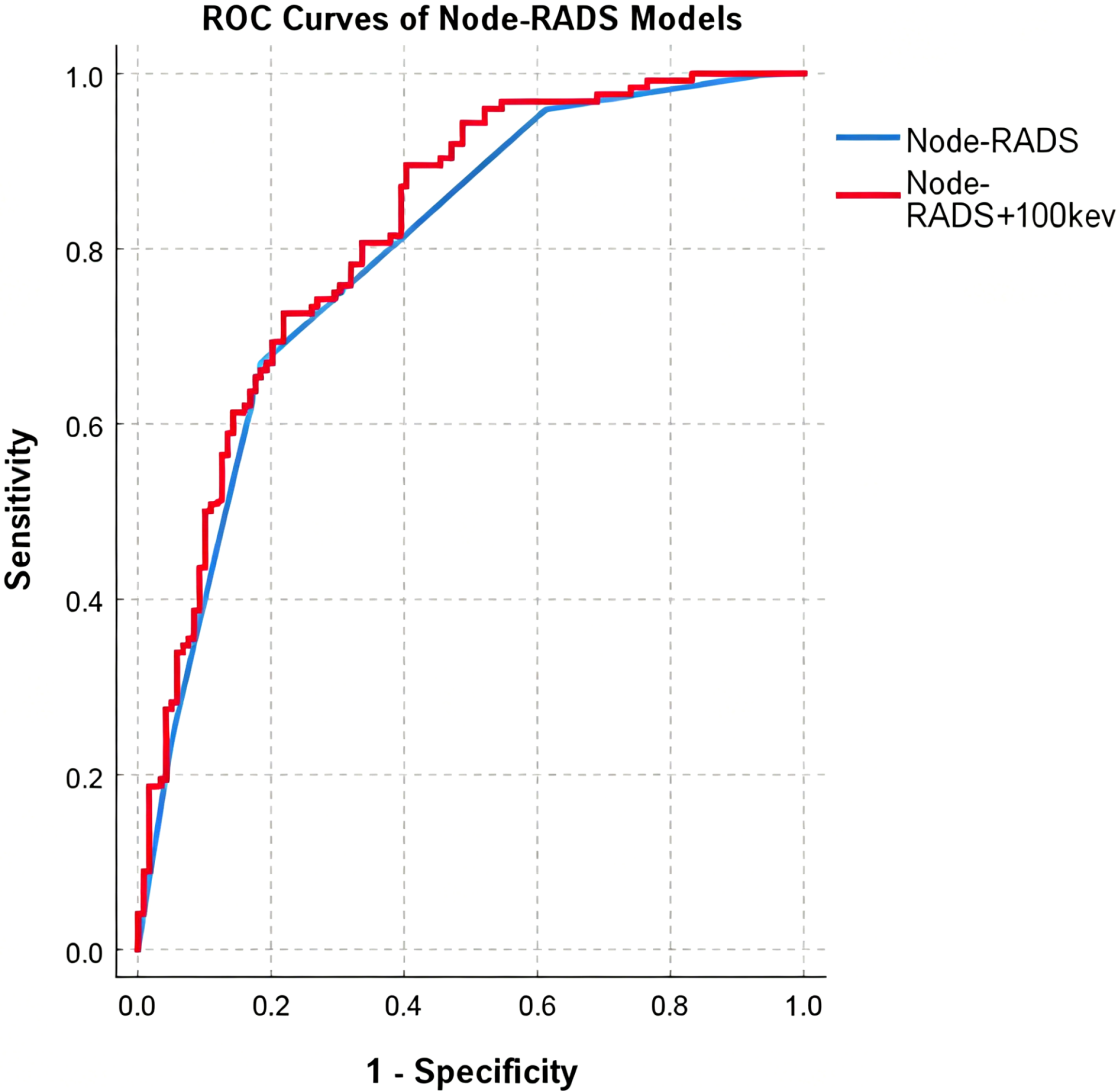
ROC curves of the logistic regression models for differentiating malignant from benign lymph nodes. The red line indicates the combined model (Node-RADS + VMI _100 keV_), and the blue line represents the baseline model (Node-RADS alone).

**Figure 5:**
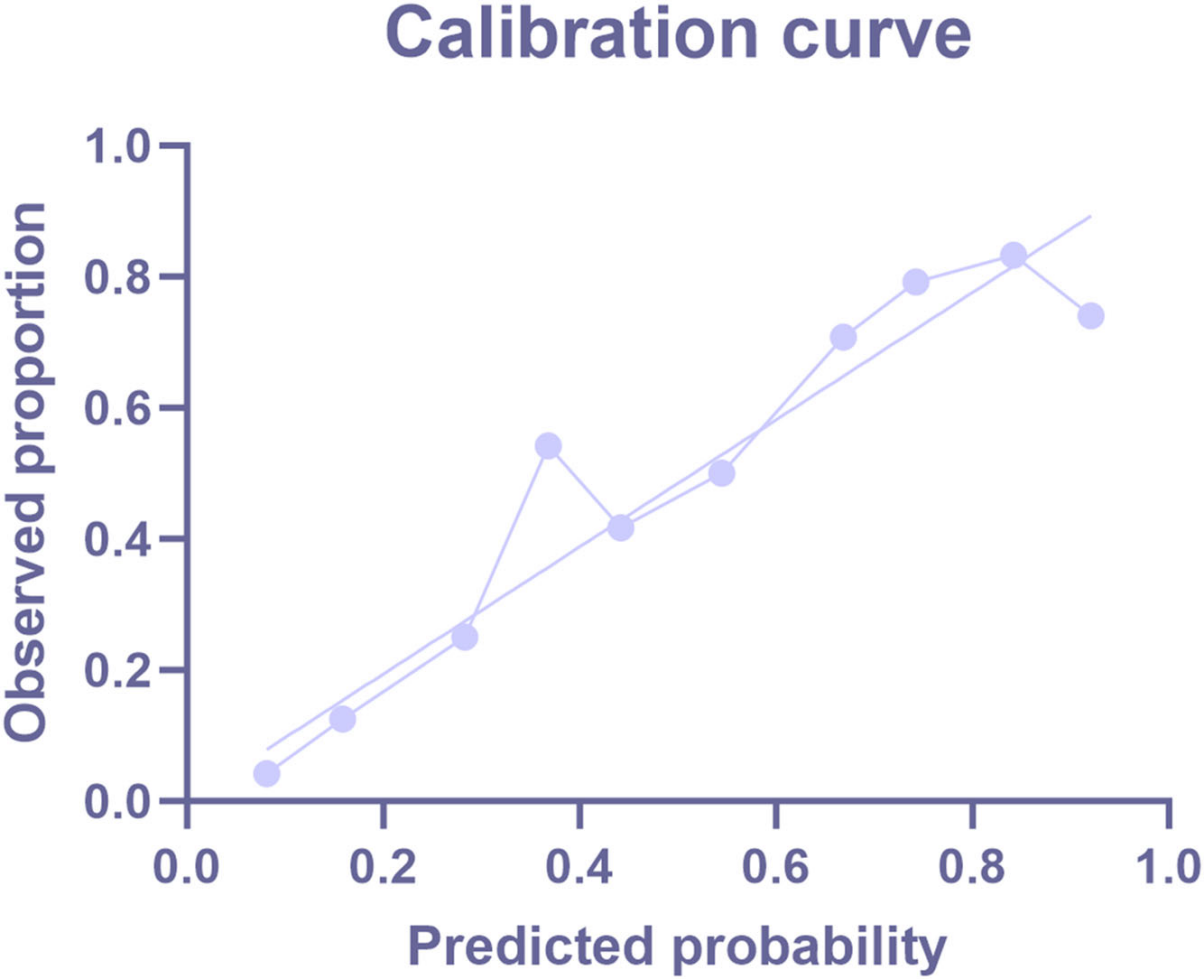
Calibration plot. Predicted versus observed probability of malignant lymph nodes.

We constructed two models and compared their performance using ROC curves (**Figure 4**). The baseline model, based on Node-RADS scores, had an AUC of 0.796 (95% CI: 0.743–0.849), sensitivity of 66.9% (95% CI: 0.579–0.751), specificity of 81.5% (95% CI: 0.733–0.881), accuracy of 74.1% (95% CI: 0.681–0.794), and a Youden’s index of 0.484. A combined model was created by incorporating the VMI _100 keV_ quantitative parameter into the baseline model, resulting in improved diagnostic performance; the AUC increased to 0.819 (95% CI: 0.766–0.872), with a sensitivity, specificity, and accuracy of 72.6%(95% CI: 0.638–0.802), 78.2% (95% CI: 0.696–0.853), and 75.3% (95% CI: 0.694–0.806), respectively, and Youden’s index of 0.508. The DeLong test showed significant differences in AUC between the two models (difference = 0.023, 95% CI: 0.002–0.043, *Z* = 2.138, *P* = 0.033). The optimal cutoff values for the baseline and combined models were 0.560 and 0.495, respectively. Bootstrap internal validation confirmed model stability, as the 95% CIs for Node-RADS (1.031–1.807, P = 0.001) and VMI 100 keV (-0.060 to -0.009, P = 0.014) did not cross zero. The combined model demonstrated good calibration (Brier score = 0.1731; Hosmer–Lemeshow P = 0.597), with excellent agreement between predicted and observed probabilities on the calibration plot (**Figure 5**). To address potential verification bias, a sensitivity analysis restricted solely to pathology-proven nodes (n = 113) was performed. Although the overall diagnostic performance slightly decreased in this clinically challenging sub-cohort, the combined model (AUC = 0.722; 95% CI: 0.622–0.822) still consistently outperformed the baseline Node-RADS model (AUC = 0.706; 95% CI: 0.606–0.807).

## 4 Discussion

Accurate assessment of lymph node status is crucial for preoperative staging, treatment planning, and prognosis evaluation in patients with common solid tumors (12–15). It also guides lymph node fine-needle aspiration biopsy, thereby reducing unnecessary procedures and extensive dissection. This study primarily found that VMI_100 keV_, a virtual monoenergetic image quantitative parameter derived from spectral CT during the venous phase, is an independent predictor of benign versus malignant lymph node enlargement in patients with common solid tumors. More importantly, the combined approach, which integrated this parameter with the Node-RADS score, showed a modest improvement in statistical discrimination compared to morphological assessment alone. The Node-RADS 1.0 defines the size, margins, and internal features of lymph nodes (16) using detailed scoring criteria, providing clinicians with a standardized framework for imaging reports. A growing body of evidence extensively supports the role of Node-RADS improves inter-reader reliability and facilitating multidisciplinary team decision-making in site-specific oncologic settings (3, 4). Our findings align with this evidence, demonstrating excellent inter-observer agreement for Node-RADS scoring (Kappa > 0.80) in our cohort. Nevertheless, routine clinical integration of such RADS-based frameworks requires further prospective and multicenter validation. However, in actual clinical practice, some early-stage micrometastatic lymph nodes receive low scores owing to the absence of typical liquefactive necrosis, whereas severe granulomatous inflammatory proliferation can result in blurred borders and, thus, high scores. Hence, a single morphological score has limited diagnostic specificity in distinguishing lymph nodes that exhibit both benign and malignant features. Therefore, combining lymph node morphological characteristics with quantitative parameters from spectral CT may provide a modest statistical improvement in discriminating malignant lymph nodes, thereby achieving complementary integration of morphology and function.

Spectral CT provides a low-energy range of 40–60 keV (17, 18), offering enhanced sensitivity to iodinated contrast agents, facilitating arterial-phase scanning and assessment of highly vascularized lesions. This study focused on lung, liver, colorectal, and breast malignancies. The metastatic lymph nodes associated with these tumors are often fibrotic or necrotic and exhibit moderate or poor blood supply (19). We employed GSI imaging during the venous phase when the contrast agent reached distribution equilibrium within the tissues. The 100 keV energy level falls within the medium-to-high energy range, effectively suppressing various artifacts and reducing image noise (20–22). Consequently, the stability and accuracy of CT value measurements are superior to those obtained at lower keV levels, which not only reduce the interference from simple iodine enhancement but may also potentially reflect differences in intrinsic tissue attenuation hypothesized to relate to the dense arrangement of tumor cells, fibrosis (23), and necrotic changes within metastatic lymph nodes. However, because no direct radiologic-pathologic spatial correlation was performed in our study, these biological interpretations remain speculative and warrant further investigation. Studies have shown that in breast and rectal cancers, VMI _100 keV_ is an integral part of multi-parameter diagnostic assessments (24, 25), and that there is a significant difference in CT values between VMI _100 keV_ during the venous phase and benign lymph nodes. The VMI _100 keV_ CT values of the malignant lymph node group were significantly lower than those of the benign lymph node group(*P* < 0.05). This finding suggests that VMI _100 keV_, a quantitative spectral CT parameter, may serve as an exploratory quantitative parameter to assist in distinguishing between benign and malignant enlarged lymph nodes in common solid tumors. However, the modest incremental statistical benefit of the combined model warrants cautious interpretation. While adding VMI 100 keV significantly improved the overall AUC, the absolute improvement was modest (from 0.796 to 0.819). Moreover, while the combination increased sensitivity (from 66.9% to 72.6%), it slightly reduced specificity (from 81.5% to 78.2%). Specifically, the gain in sensitivity comes at the expense of a reduction in specificity. In clinical practice, this increase in false-positive classifications could potentially lead to overstaging, unnecessary biopsies, or overtreatment. Given the absence of formal decision-analytic or cost-benefit assessments in the present study, the actual net clinical benefit of this trade-off remains uncertain. Therefore, the practical clinical benefit of adding VMI 100 keV to routine Node-RADS assessment remains uncertain at this stage. These preliminary exploratory findings do not yet support modification of routine clinical assessment workflows without further prospective validation.

This study had certain limitations. First, the retrospective single-center design inherently limits the generalizability of our findings. Although internal bootstrap validation was performed to assess model optimism, this technique cannot substitute for external validation in an independent cohort, which remains essential to confirm clinical applicability. Furthermore, although multicollinearity was assessed and an iterative selection strategy was applied, residual instability due to correlations among spectral parameters cannot be excluded. Second, different tumor types (lung, liver, colorectal, and breast cancers) were pooled in the cohort to evaluate the overall exploratory performance of the combined approach. While this approach demonstrates the general applicability of VMI 100 keV across common solid tumors, these malignancies are biologically heterogeneous and exhibit distinct metastatic patterns, nodal microenvironments, and enhancement characteristics. Notably, this study was primarily designed as an initial proof-of-concept rather than being statistically powered, aiming to establish individualized diagnostic thresholds tailored to each specific primary tumor. Therefore, applying a unified diagnostic threshold across varying tumors should be approached with caution. Moreover, future site-specific studies are required to fine-tune and validate these parameters for individual cancer types. Third, the nature of some nodes in the full cohort was determined via imaging follow-up rather than pathology; therefore, misclassification bias may have been introduced despite our rigorous longitudinal assessment criteria. To partially address this concern, we performed a sensitivity analysis on the pathology-only subset; however, the relatively small sample size of this subgroup may limit its statistical power. Therefore, we used the mixed cohort as a practical approach to maintain overall model stability. Fourth, to ensure accurate ROI placement, we restricted inclusion to lymph nodes with a short-axis diameter of primarily ≥ 5 mm. While this approach enables the evaluation of sub-centimeter nodes, it inherently influences the baseline size scores in Node-RADS. Therefore, the applicability of this approach for microscopic metastases (< 5 mm) remains unknown.

In summary, these preliminary exploratory observations suggest that combining spectral CT-based VMI 100 keV with Node-RADS scores during the venous phase provides a modest statistical improvement in evaluating lymph node metastasis in common solid malignancies. However, considering the reduction in specificity, the practical net clinical benefit of this approach remains uncertain. This proof-of-concept framework requires external validation in independent cohorts and formal clinical utility analyses before any modification of routine radiologic assessment workflows can be considered.

## Data Availability

The original contributions presented in the study are included in the article/supplementary material, further inquiries can be directed to the corresponding author/s.
